# CRISPR-Cas9 Approach Constructing Cellulase *sestc-*Engineered *Saccharomyces cerevisiae* for the Production of Orange Peel Ethanol

**DOI:** 10.3389/fmicb.2018.02436

**Published:** 2018-10-10

**Authors:** Peizhou Yang, Yun Wu, Zhi Zheng, Lili Cao, Xingxing Zhu, Dongdong Mu, Shaotong Jiang

**Affiliations:** College of Food and Biological Engineering, Anhui Key Laboratory of Intensive Processing of Agricultural Products, Hefei University of Technology, Hefei, China

**Keywords:** biomass ethanol, cellulase, *Saccharomyces cerevisiae*, CRISPR-Cas9, orange peel, sestc

## Abstract

The development of lignocellulosic bioethanol plays an important role in the substitution of petrochemical energy and high-value utilization of agricultural wastes. The safe and stable expression of cellulase gene *sestc* was achieved by applying the clustered regularly interspaced short palindromic repeats-Cas9 approach to the integration of *sestc* expression cassette containing *Agaricus biporus* glyceraldehyde-3-phosphate-dehydrogenase gene (*gpd*) promoter in the *Saccharomyces cerevisiae* chromosome. The target insertion site was found to be located in the *S. cerevisiae* hexokinase 2 by designing a gRNA expression vector. The recombinant SESTC protein exhibited a size of approximately 44 kDa in the engineered *S. cerevisiae*. By using orange peel as the fermentation substrate, the filter paper, endo-1,4-β-glucanase, exo-1,4-β-glucanase activities of the transformants were 1.06, 337.42, and 1.36 U/mL, which were 35.3-fold, 23.03-fold, and 17-fold higher than those from wild-type *S. cerevisiae*, respectively. After 6 h treatment, approximately 20 g/L glucose was obtained. Under anaerobic conditions the highest ethanol concentration reached 7.53 g/L after 48 h fermentation and was 37.7-fold higher than that of wild-type *S. cerevisiae* (0.2 g/L). The engineered strains may provide a valuable material for the development of lignocellulosic ethanol.

## Introduction

Bioethanol, as a promising alternative to petroleum resources, can be produced from lignocellulosic biomass and starch-rich plants (Kou et al., 2017). The development of lignocellulosic ethanol could be more promising in the future than food crop ethanol considering its sustainable, renewable, and environmentally friendly features (Cai et al., 2016). The conversion of lignocellulosic materials requires three key steps, namely, biomass pretreatment, saccharification, and fermentation (Chang et al., 2013). Pretreatment changes the physical and chemical properties of the raw materials. Saccharification produces fermentable sugars from cellulosic materials via enzymatic degradation, acidolysis, and ionic hydrolysis (Zhao et al., 2018). Fermentation converts D-glucose and other monosaccharides into ethanol by using microorganisms. A highly efficient transformation of lignocellulosic materials requires the integration of these three processing technologies (Koppram et al., 2014).

Orange is an important fruit around the world. During the orange processing, orange peel is removed from the flesh and discarded as a waste (Pandiarajan et al., 2018). The wasted orange peel generally decays and severely pollutes the atmosphere, soil, and water. The rational use of wasted orange peel has important value and significance (Rehan et al., 2018). The compositions of dry orange peel (w/w) are 17.5% of cellulose, 8.6% of hemicellulose, 25.4% of pectin, 6.73% of protein, 7.7% of moisture, 4.4% of ash (Miran et al., 2016), and 0.85% of lignin (Kantar et al., 2018). The cellulose can be converted into D-glucose via the hydrolysis of cellulase. Due to its lower lignin content, cellulase is more readily accessible to cellulose (Gao et al., 2014). In this study, orange peel was adopted to produce ethanol using the cellulase-engineering *Saccharomyces cerevisiae*. By *in situ* saccharification and fermentation, the production of ethanol from orange peel was investigated. Admittedly, orange peel is a representative of lignocellulosic materials. Other lignocellulosic materials such as corn straw and rice straw would be further investigated for the production of ethanol.

As an engineered host strain, *S. cerevisiae* can effectively produce ethanol and express heterologous proteins (Romanos et al., 1992; Ruohonen et al., 1997). However, the extremely low activities of cellulase produced by *S. cerevisiae* severely limit the conversion of lignocellulosic materials into fermentable sugars (Yang et al., 2018). Currently, the high cost of commercial cellulase considerably reduces the competitiveness of bioethanol in the market compared with fossil energy (Aswathy et al., 2010). The construction of engineered *S. cerevisiae* expressing cellulase is an important approach to degrading lignocellulosic materials (Kroukamp et al., 2018).

Previous reports on the engineered strain of cellulase genes mainly focused on the transformation of expression vectors embracing antibiotic resistance genes (Yang et al., 2016a). These expression vectors are located in the cytoplasm and are easily lost during cell proliferation. The addition of antibiotics raises the production cost, contaminates the broth, and reduces the product purity (Solange et al., 2010). Therefore, the integration of the cellulase gene into the *S. cerevisiae* genome without the addition of antibiotics should be an economic, safe, and feasible solution to producing bioethanol (Lee et al., 2017). As an RNA-mediated adaptive immune system, clustered regularly interspaced short palindromic repeats-Cas9 (CRISPR-Cas9) has been developed to achieve gene editing through its RNA-guided endonuclease activity (Singh et al., 2017). A segment of DNA is integrated into the host genome by an RNA-mediated approach (Mali et al., 2013). The integrated heterogenous DNA can be sustainably preserved in the engineered strain without antibiotic resistance genes (Jinek et al., 2014). Therefore, CRISPR-Cas9 is an effective approach to construct engineered polyploid industrial yeast and fungal strains (Liu et al., 2017; Lian et al., 2018).

The cellulase gene used in this study was isolated from the stomach tissue of *Ampullaria gigas* Spix (Yang et al., 2018). The gene encoding protein possesses three kinds of cellulase activity, namely, endo-1,4-β-glucanase (EG), exo-1,4-β-glucanase (CBH), and β-xylanase, and was named single-enzyme-system-three-cellulase (*sestc*). In this study, the expression cassette carrying a cellulase gene from *A. gigas* Spix was integrated into the *S. cerevisiae* chromosome by using a CRISPR-Cas9-based approach. The novel engineered strains can simultaneously express SESTC cellulase and produce ethanol by consuming the glucose obtained from the SESTC enzymatic hydrolysis. All the processes of SESTC expression, lignocellulosic saccharification, and ethanol production were investigated without the addition of any antibiotics. This study is valuable for developing lignocellulosic ethanol with a lignocellulosic material as the carbon source.

## Materials and Methods

### Strains, HXK2-gRNA, Cas9-NAT, and *sestc* Expression Cassette

The *S. cerevisiae* used in this study was derived from diploid industrial yeast. The Cas9 expression cassette was carried by a yeast single-copy episomal plasmid. The Cas9-NAT plasmid carrying selectable markers nourseothricin and bacterial resistance ampicillin was from Addgene and is reconstructed from the vector backbone pRS414-TEF1p-Cas9-CYC1t (Zhang et al., 2014; Chin et al., 2016). The gRNA expression vector for targeting *S. cerevisiae* hexokinase 2 gene (HXK2-gRNA) was constructed by amplifying gRNA-trp-HYB, which is preserved in Addgene, and using the primers shown in **Table 1**.

**Table 1:** Primers and target sequences of *S. cerevisiae* HXK2-gRNA.

Product features of PCR amplification	Upstream primers	Downstream primers
*L. edodes* *gpd* promoter, 617 bp, designed according to Genbanks Accession GQ457137	5′-tttatgctggttatctgagcg-3′	5′-ttattcaagcagtcaatggat-3′
*sestc* cassettes embraced by the backbone of pBluescript II KS(-)	5′-gtaaaacgacggccagt-3′	5′-aacagctatgaccatg-3′
Amplification for *S. cerevisiae* hexokinase 2 deletion HXK2-gRNA cassette	5′-ctcattttggaacaagtcatgttttagagctagaaatagcaag-3′	5′-atgacttgttccaaaatgaggatcatttatcttt cactgcgga-3′
PCR and RT-PCR identification	5′-ttgccctctgagtgtcgctc-3′	5′-tcgacgacgcttcagtcaagc-3′
Recognition sequences of *S. cerevisiae* hexokinase 2 gRNA and two side sequences from *S. cerevisiae*	tctttgaaaagataatgtatgattatgctttcactcatatttatacagaaacttgatgttttctttcgagtatatacaaggtgattacatgtacgtttgaagtacaactctagattttgtagtgccctcttgggctagcggtaaaggtgcgca ttttttcacaccctacaatgttctgttcaaaagattttggtcaaacgctgtagaagtgaaagttggtgcgcatgtttcggcgttcgaaacttctccg cagtgaaagataaatgatcctcattttggaacaagtcatgttttagagctagaaatagcaagttaaaataaggctagtccgttatcaacttgaaa aagtggcaccgagtcggtggtgctttttttgttttttatgtct	

*The underline indicated the gRNA target sequences of the *S. cerevisiae* hexokinase 2 gene based on the sequences of *S. cerevisiae* hexokinase 2 (NCBI Genbanks ID:852639)*.

The *sestc* expression cassette for cellulase gene expression was isolated as follows. (1) *Lentinula edodes* glyceraldehyde-3-phosphate dehydrogenase (*gpd*) gene promoter was isolated from *L. edodes* genome by using *L. edodes* *gpd* promoter primers. The upstream and downstream regions of the *L. edodes gpd* promoter contained the restriction enzyme cutting sites of *Sac* I and *Spe* I. The *L. edodes gpd* promoter was inserted into the pBluescript II KS(-) plasmid by the double-enzyme digestion of *Sac* I, *Spe* I, and ligase. (2) The *sestc* fragment containing *Spe* I and *BsrG* I cutting sites was integrated into the plasmid containing the *L. edodes gpd* promoter. (3) A pair of primers were designed to amplify a complete *sestc* cassette embraced by the backbone of pBluescript II KS(-). The isolated *sestc* cassette contained the *L. edodes gpd* promoter, *sestc*, and the terminator.

### CRISPR-Cas9 Approach Integrating the *sestc* Expression Cassette

**FIGURE 1:**
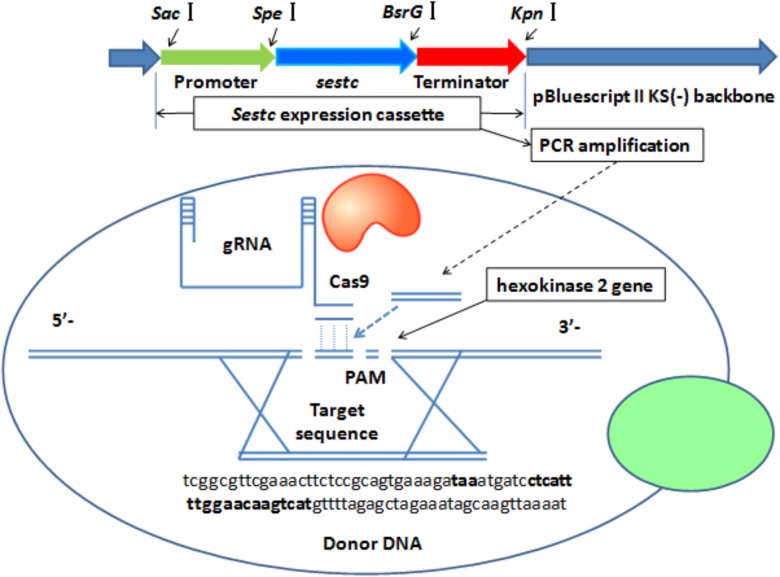
CRISPR-Cas-directed gene disruption for the integration of *sestc* expression cassettes in the *Saccharomyces cerevisiae* genome. 5′-Tcggcgttcgaaacttctccgcagtgaaagataaatgatcctcattttggaacaagtcatgttttagagctagaaatagcaagttaaaat-3′ were the partial sequences of sgRNA expression vector. Both the left and right sequences, respectively located at bold font taa and 5′-ctcattttggaacaagtcat-3′ are from *S. cerevisiae* chromosome. 5′-Ctcattttggaacaagtcat-3′ was designed as a 20-nt sgRNA according to *S. cerevisiae* hexokinase 2 gene. Termination codon taa was designed to stop the gene expression of its upstream sequences and start the synthesis of 20-nt sgRNA. *Sestc* expression cassette was composed with promoter gene, *sestc*, and terminator. The cassette amplified *via* PCR approach was inserted into the locus of *S. cerevisiae* genome cut by Cas9 Nuclease.

The *sestc* expression cassette was integrated into the *S. cerevisiae* genome by knocking out the *hxk*2 gene via CRISPR-Cas9-directed gene disruption (**Figure 1**). A double-strand break was formed at the gRNA target sequence. As a donor DNA, the *sestc* cassette was inserted into the *S. cerevisiae* chromosome in the double-strand break. The specific target sequence of the sgRNA (5′-ctcattttggaacaagtcatcgg-3′) for *hxk*2 deletion was designed using an online software^1^. The *sestc* gene cassette was inserted into the *S. cerevisiae* chromosome by using the two-step CRISPR-Cas9 approach. (1) The Cas9 plasmid was integrated into the *S. cerevisiae*. The transformation of the Cas9 plasmid containing a nourseothricin resistance gene was performed by the LiAc-PEG method (Schiestl, 2007). Approximately 50 μL of the transformation solution was cultivated on YPD agar plates containing 100 μg/mL nourseothricin at 30°C for 2 days. The true transformants were named *S. cerevisiae*-Cas9. (2) The HXK2-gRNA expression plasmid containing a hygromycin B resistance gene and *sestc* expression cassette were synchronously transformed into *S. cerevisiae*-Cas9 by the LiA-PEG-mediated method (Schiestl, 2007). The transformation solution was cultivated on YPD agar plates containing 80 μg/mL nourseothricin and 200 μg/mL hygromycin B at 30°C for 2 days. The putative transformants were further identified at molecular and protein levels.

### RT-PCR Identification and Protein Analysis of Recombinant *S. cerevisiae*

Before the identification, the putative transformants were cultured in liquid YPD media at 30°C and a rotational speed of 280 rpm for 3 days. The monoclonal colonies without nourseothricin and hygromycin resistance were selected for further identification. The *S. cerevisiae* transformant RNA was extracted using a TransZol Plant extraction kit from TransGen Biotech Co., Ltd. EasyScript First-Strand cDNA Synthesis SuperMix from TransGen Biotech Co., Ltd. was used to synthesize the complementary DNA, with the transformant RNA as the template. The designed primers based on the *sestc* sequences were used to amplify *sestc*, with wild-type *S. cerevisiae* as the control. The total proteins of the engineered and wild-type *S. cerevisiae* were analyzed by the sodium dodecyl sulfate–polyacrylamide gel electrophoresis (SDS-PAGE) approach.

### Cellulase Expression, Saccharification, and Ethanol Production

Exactly 20 mL of 20 OD_600_ yeast cells/mL was inoculated into a 500 mL Erlenmeyer flask loaded with 200 mL of YP medium and 10 g of oven-dried orange peel powders. After fermentation at 30°C and 180 rpm for 60 h, the cellulase activities were measured using the dinitrosalicylic acid method (Ghose, 1987). The total cellulase activity (filter paper activity, FPA), endo-β-1-4-glucanase (CMCase, EG), and exo-β-1-4-glucanase (cellobiohydrolase, CBH) were measured using Whatman grade 1 filter paper (50 mg, 1 cm × 6 cm), 0.51% CMC–Na (w/v), and 1% microcrystalline cellulose (w/v) as catalytic substrates, respectively. The saccharification process was conducted by incubating the fermentation solution at 37°C for 6 h. Subsequently, the temperature was reduced to 30°C for ethanol production under anaerobic conditions. The ethanol concentration was measured using gas chromatography with the following parameters: Agilent DB-624, FID detector, 50°C column temperature, 250°C detector temperature, 175°C injection temperature, and 1 μL injection volume. High-performance liquid chromatography (HPLC) was used to measure the content of glucose (Zhang et al., 2016).

## Results

### Cotransformation of Cas9-NAT, HXK2-gRNA, and *sestc* Expression Cassette

Cas9-NAT, HXK2-gRNA, and the *sestc* expression cassette were successively transformed into wild-type *S. cerevisiae*. After the transformation, the solution mixture was cultured on solid YPD media, which contained two antibiotics. The transformation rate of the *sestc* expression cassette was 37.32 ± 4.23 colonies/μg *sestc* expression cassettes. No colony was observed on the plates of the two negative controls. The screened colonies were used for further identification.

### PCR, RT-PCR, and SDS-PAGE Identification

**FIGURE 2:**
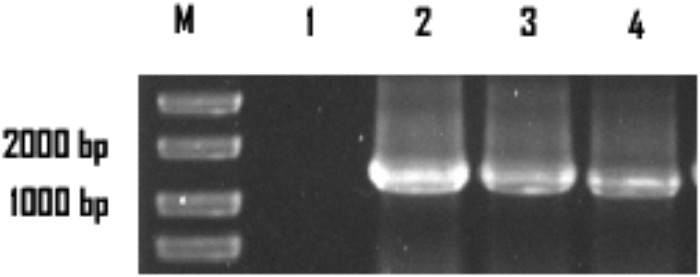
RT-PCR approach identifying the *sestc* *S. cerevisiae* transformants. Lane M marker; lane 1 wild-type *S. cerevisiae*; lane 2 the positive control; lane 3,4 the putative transformants.

**FIGURE 3:**
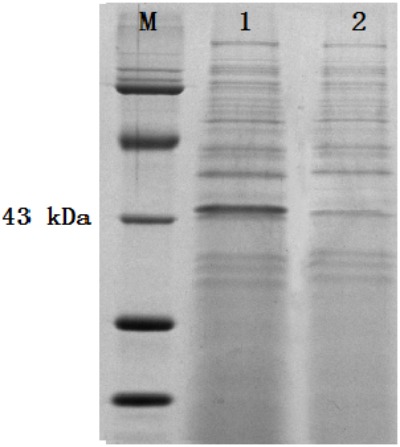
SDS-PAGE approach analyzing the total protein profiles of the identified transformants. Lane M protein marker; lane 1 *sestc* *S. cerevisiae* transformant; lane 2 wild type *S. cerevisiae.*

The colonies that lost both antibiotics were used for PCR identification by using the primers for *sestc* amplification (**Table 1**). The sizes of the putative transformant DNA bands were similar to those of the positive control. No band existed in the negative control lane (figure omitted). The positive transformants identified by PCR were further identified by the RT-PCR approach. The sizes of the DNA bands from the transformants were as expected. No DNA band existed in the wild-type *S. cerevisiae* lane (**Figure 2**). The SDS-PAGE approach was used to analyze the protein profiles of the identified transformants in comparison with the wild type *S. cerevisiae* (**Figure 3**). SESTC was approximately 44 kDa in size. Therefore, the *sestc* gene was effectively expressed in the engineered *S. cerevisiae*.

### Activity Profiles of Cellulase and Saccharification for Glucose Release

**FIGURE 4:**
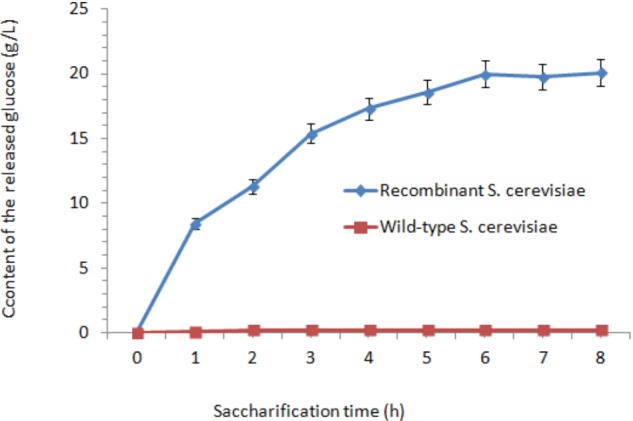
Effect of saccharification time on the content of released glucose.

The cellulase activities in wild-type *S. cerevisiae* were lower than those in the transformants. After fermentation for 48 h, the cellulase activities reached their peaks. The highest FPA, EG, and CBH were 1.06, 337.42, and 1.36 U/mL, which were 35.3-fold, 23.03-fold, and 17-fold higher than those in wild-type *S. cerevisiae*, respectively. After incubation at 37°C for 18 h, the content of the released glucose was measured. After 6 h treatment, the concentration of glucose reached its peak (20 g/L) (**Figure 4**). Afterward, the released amount of glucose nearly did not increase when treated for 6–18 h. As the control, nearly no glucose was released from the orange peel, as determined by the HPLC method.

### Anaerobic Fermentation for Ethanol Production

**FIGURE 5:**
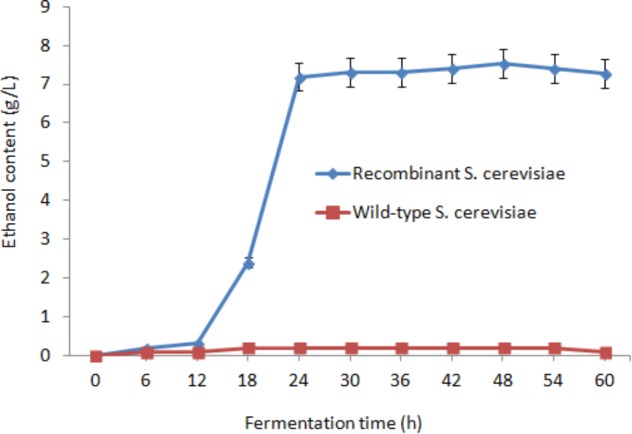
Effect of fermentation time on ethanol content.

After saccharification, the fermentation mixture containing 15 OD_600_ of the recombinant *S. cerevisiae* cells, 20 g/L glucose, residual lignocellulose, and culture medium was treated at 30°C under anaerobic conditions (**Figure 5**). After 24 h fermentation, the ethanol concentration reached 7.19 g/L. Subsequently, the growth trend of ethanol content gradually decreased. The highest ethanol concentration reached 7.53 g/L after 48 h fermentation and was 37.7-fold higher than that of wild-type *S. cerevisiae* (0.2 g/L). The conversion rate of ethanol was 0.377 g/g glucose and 0.151 g/g dry orange peel.

## Discussion

Lignocellulosic ethanol is an ideal alternative to petrochemical energy. The direct application of lignocellulosic materials can reduce the product cost. The reported materials were mainly from corn stover (Zhao et al., 2017), rice straw (Yu and Li, 2015), spruce (Stenberg et al., 2000), switchgrass (Wu et al., 2018), *Miscanthus* ×*giganteus* (Scordia et al., 2013; **Table 2**). Compared with these lignocellulosic materials, orange peel showed benefits, such as low lignin content, easy decomposition, loose structure, and high sugar content. In addition, orange peel ethanol did not require other treatments, such as steam-, acid-, and alkali-based treatments. The construction of cellulase gene-engineered *S. cerevisiae* was an effective approach to achieve *in situ* saccharification and ethanol production. *S. cerevisiae* coexpressing cellulase/cellodextrin and *Trichoderma viride* EG3/BGL1 produced ethanol contents of 4.3 g/L (Yamada et al., 2013) and 4.63 g/L (Gong et al., 2014). *S. cerevisiae* expressing *Aspergillus aculeatus* β-glucosidase achieved an ethanol content of 15 g/L (Treebupachatsakul et al., 2016). *S. cerevisiae* expressing Cel3A, Cel7A, and Cel5A achieved an ethanol content of 10% (w/v) (Davison et al., 2016). The current study indicated that the final ethanol concentration reached 7.53 g/L, and the conversion rates of ethanol were 0.377 g/g glucose and 0.151 g/g dry orange peel. These indices approached or exceeded those previously reported (Yamada et al., 2013; Gong et al., 2014; Yu and Li, 2015; Wu et al., 2018).

**Table 2:** Reported production of lignocellulosic ethanol.

Strains	Lignocellulosic materials	Ethanol yields
*Sestc* engineering *S. cerevisiae*	Orange peel	7.53 g/L, 0.151 g/g orange peel, this study
Recombinant *S. cerevisiae* and adapted *P. stipistis*	Steam exploded corn stover (SECS)	27 g/111.51 g SECS (Zhao et al., 2017)
*Gracilibacillus sp* SK1	Corn stover and rice straw	0.186 g/g dry substrate (Yu and Li, 2015)
Beta-glucosidase secretion in *S. cerevisiae*	Acid pretreated corncob	89% higher than the control strain (Tang et al., 2013)
*S. cerevisiae* coexpressing cellulase/cellodextrin	Phosphoric acid swollen cellulose	4.3 g/L (Yamada et al., 2013)
Commercial enzymes and *S. cerevisiae*	Spruce pretreated by steam	68% of the theoretical yield (Stenberg et al., 2000)
*S. cerevisiae* expressing Cel3A, Cel7A, and Cel5A	Lignocellulosic substrates	10% w/v (Davison et al., 2016)
*S. cerevisiae* coexpressing *TrCel3B*, and *AaBGL1*	Lignocellulosic biomass	15 g/L (Treebupachatsakul et al., 2016)
*S. cerevisiae* coexpressing *T. viride* EG3 and BGL1	Carboxymethyl cellulose	4.63 g/L (Gong et al., 2014)
*E. coli* strain SL100	Switchgrass pretreated with phosphoric acid	21.2 +/- 0.3 g/L (Wu et al., 2018)
*Scheffersomyces stipitis* CBS 6054	Miscanthus × gigantueus pretreated by steam	12.1 g/L (Scordia et al., 2013)

The high-value utilization of wastes from agricultural products reduces environmental pollution (Tetsuya et al., 2013). Previous studies on cellulase-engineered *S. cerevisiae* focused on the gene expression in the cytoplasm by using vectors that carry antibiotic resistance genes, such as nourseothricin and hygromycin B resistance genes (Koppram et al., 2014; Yang et al., 2016b). In this study, the cellulase *sestc* cassette was integrated into the *S. cerevisiae* genome by using CRISPR-Cas9 technology to achieve the stable expression of the lignocellulolytic enzyme in *S. cerevisiae*. The *sestc* gene can be expressed at the level of the *S. cerevisiae* genome with the *gpd* promoter by using orange peel as the fermentation substrate. This study provided a valuable reference on cellulase *sestc*, particularly its expression in cellulase and its role in ethanol production with lignocellulosic materials. However, the cellulase *sestc-*engineered *S. cerevisiae* still exhibited problems. The lack of β-glucosidase activity in the *sestc-*engineered strains caused insufficient substrate hydrolysis. The heterologous expression of β-glucosidase gene in the *sestc*-engineered strains is a reasonable solution; in addition, the supplement to β-glucosidase in the solution containing SESTC is also an effective strategy.

## Conclusion

Crushed orange peel powder was used to produce ethanol via saccharification and fermentation of *sestc-engineered* *S. cerevisiae*. The highest ethanol concentration was 7.53 g/L after 48 h fermentation. This value was 37.7-fold higher than that of wild-type *S. cerevisiae*. The conversion rates of ethanol were 0.377 g/g glucose and 0.151 g/g dry orange peel. This study provided an effective approach to integrate cellulase *sestc* cassettes into the *S. cerevisiae* genome via the CRISPR-Cas9 approach. The constructed engineered *S. cerevisiae* can be applied to biomass ethanol production.

## Author Contributions

PY proposed the research and wrote the manuscript. YW performed the experiments. ZZ analyzed the data. XZ made the maps. SJ designed the scheme. DM wrote the discussion section. LC measured the ethanol content.

## Conflict of Interest Statement

The authors declare that the research was conducted in the absence of any commercial or financial relationships that could be construed as a potential conflict of interest.

## Footnotes

1 http://chopchop.cbu.uib.no/
